# Molecular dynamics simulations on aqueous two-phase systems - Single PEG-molecules in solution

**DOI:** 10.1186/2046-1682-5-14

**Published:** 2012-08-08

**Authors:** Stefan A Oelmeier, Florian Dismer, Jürgen Hubbuch

**Affiliations:** 1 Karlsruhe Institute of Technology (KIT), Institute of Process Engineering in Life Sciences, Section IV: Biomolecular Separation Engineering, Karlsruhe, Germany

## Abstract

**Background:**

Molecular Dynamics (MD) simulations are a promising tool to generate molecular understanding of processes related to the purification of proteins. Polyethylene glycols (PEG) of various length are commonly used in the production and purification of proteins. The molecular mechanisms behind PEG driven precipitation, aqueous two-phase formation or the effects of PEGylation are however still poorly understood.

**Results:**

In this paper, we ran MD simulations of single PEG molecules of variable length in explicitly simulated water. The resulting structures are in good agreement with experimentally determined 3D structures of PEG. The increase in surface hydrophobicity of PEG of longer chain length could be explained on an atomic scale. PEG-water interactions as well as aqueous two-phase formation in the presence of PO_4_ were found to be correlated to PEG surface hydrophobicity.

**Conclusions:**

We were able to show that the taken MD simulation approach is capable of generating both structural data as well as molecule descriptors in agreement with experimental data. Thus, we are confident of having a good *in silico* representation of PEG.

## Background

Polyethylene glycol (PEG) is among the most commonly used chemicals in protein purification processes. Its use ranges from precipitating agent [1,2], phase forming component in liquid-liquid extraction [3-6], displacer in hydrophobic interaction chromatography [7] to potential additive in protein formulation [8,9] and drug modifying agent [10,11]. Despite its frequent application, the exact nature of the polymer solvent interactions as well as the structural dynamics in solution governing its role in protein purification processes remain unclear.

One approach to understand the nature of molecules on an atomic scale are molecular dynamics (MD) simulations. MD simulations have proven a useful tool to understand the binding and elution behavior of proteins in ion exchange chromatography [12]. Its results could be correlated to experimental data and thus used predictively. Besides HTS process development methodologies, MD simulations represent another promising approach to speed up process development in protein purification.

Several molecular dynamics studies have been performed focusing on the tertiary structure of polyethylene glycol or polyethylene oxide in aqueous solutions. Lee *et al.* in 2008 [13] used a revision of the CHARMM ether force field (C35) [14] to establish a molecular understanding of the relation between hydrodynamic radius and radius of gyration of PEG, which differs in behavior from polymer theory under certain conditions. They extended their study in 2009 [15] by using a coarse grained model and were thus able to extend the simulated timespan from 20 to 800 ns. These studies were again focused on investigating the hydrodynamic properties of the polymer molecules, their hydrodynamic radius in relation to their radius of gyration and the coherence of the simulation results with polymer theory. One major goal was to understand - on a molecular scale - the influence of the three dimensional structure of polymer molecules in solution on macroscopic, hydrodynamic properties of these solutions e.g. viscosity. Other studies, such as the one conducted by Borodin *et al.* in 2001 [16] are geared towards polymer dynamics of pure polymers and the effect of added water. One main goal of this study was again to understand the physicochemical properties of polymer solutions such as viscosity.

Tasaki *et al.* performed MD simulation of a single PEG molecule of 722 Da in 1996 [17] focused more on the secondary structure and polymer-water interaction. While their study yielded interesting insights into the 3D structure of single PEG molecules in solution, the advance in computational power since their publication now allows for longer simulations of larger PEG molecules. Another, more recent, MD simulation study using a PEG-derivate focused the importance of choosing a proper force field [18] to obtain secondary structure results in agreement with experimental data. One force field, not tested in their study, but commonly used for protein simulations, is the amber03 force field [19]. While this force field is designated to proteins, a self parameterizing algorithm can be applied to adjust for non proteinaceous molecules [20]. The advantage of using a force field designated to proteins is that combined MD simulations of proteins and PEG could be readily run, once it is established that the self parameterizing algorithm yield reasonable results for PEG. In order to verify the structural data resulting from such MD simulations, experimentally determined structures are needed. There have been several reports of 3D structural data of PEG [21] with the recent addition of a high quality crystallographic record [22].

As stated above, MD simulations focused on the secondary structure of single PEG molecules of fixed molecular weight as well as studies concentrating on the three dimensional structure and its implications for macroscopic solution properties have been conducted. Simulations of larger PEG molecules and an investigation of the effects of polymer molecular weight on properties of PEG such as secondary structure, surface hydrophobicity and H-bond network with the solvent are lacking. These properties most likely govern the characteristics of PEG molecules in protein purification, thus their deeper understanding on a molecular scale would be beneficial. PEG polymer molecular weight influences the effect PEG has on protein solubility or aqueous two-phase formation. To understand the influence of molecular weight of PEG molecules on their structural and surface characteristics on an atomic scale would thus be valuable.

This study’s first aim is to confirm the validity of all-atom molecular dynamics simulations of PEG in water based on the amber03 force field by comparing the resulting structure to the recently published crystallographic data and previous MD results. Results from 10-30 ns long MD simulations of PEG molecules ranging in molecular weight from 300 Da to 3500 Da are shown. This study puts a focus on the effect PEG chain length has on geometric parameters, secondary structure, polymer surface characteristics and polymer solvent interactions. We aim to understand the influence on PEG molecular weight on polymer properties important for its use in protein purification. Correlations to experimental data on the phase forming behavior of 4 different PEGs in the presence of phosphate are given. Correlation between solvent polarity determined using a solvatochromic dye to hydrophobicity determined via MD simulations are shown.

## Methods

### Molecular dynamics simulation software

Molecular dynamics simulations were performed using the Yasara Structure software package [23], version 10.2.1. The software was installed in a cluster computer environment running Suse Linux Enterprise 10 as operating system. Each simulation was run using a single node of the cluster computer. Each node was equipped with 2 Intel Xeon Quad Core (X5355) processors at 2.66 GHz and 16GB local memory (2GB per processor core).

### Force field

The software package used for the simulations employs an automatic parametrization algorithm (termed “AutoSMILES”) to generate force field parameters for unknown structures. The method is described in detail in [19,20]. This method was used to generate force field parameters for the polymer molecules. Van der Vaals forces were truncated at a cutoff of 10 Å. Long range Coulomb interactions were calculated using the Particle Mesh Ewald algorithm detailed by Essmann *et. al*[24]. Grid point for the PME evaluation were evenly spaced in each dimension. The amber03 force field [25] was used for all molecular dynamics simulations. As water model, TIP3P was used. The amber03 force field was chosen as following studies were to include protein molecules in the simulation and amber03 is a well established force field for protein simulations.

### MD simulation protocol

All-atom molecular dynamics simulations were run for PEGs with subunit numbers (n) between 6 and 81 (6, 7,…, 21 subunits; 21, 23,…, 41 subunits, and 41, 45,…, 81 subunits). Each simulation consisted of a single PEG molecule surrounded by water molecules. Number of atoms simulated ranged from 1572 for n=6 to 17211 for n=81. PEG molecules were imported into the simulation software using an OpenBabel plug-in and SMILES file format. Thus, each simulation initially contained a perfectly linear PEG molecule with all geometric parameters set to standard values. A simulation box was put around the PEG molecule with a distance of 5 nm. The simulation box’s size ranged from 28.33 x 24.23 x 23.59 Å for n=6 to 309.14 x 23.59 x 23.59 Å for n=81. Boundary conditions were set to periodic. Water density was set to 1 g/cc, temperature to 298K. The simulation box was (automatically) filled with water molecules to the set density and a short steepest decent energy minimization was run. Finally, a 5 ns long molecular dynamics simulation was started and snapshots of the simulation taken every 5 ps. Table 1 summarizes the parameters set for these simulations.

**Table 1:** MD simulation parameter used in this study

**Parameter**	**Value**
Force-Field	Amber03 [25,35,36]
Wall boundaries	periodic
Simulation time	10-30 ns
Snapshot interval	5 ps
Density [g/l]	1.0
pH	7.0
Temperature control	rescale velocities [37,38]

### MD snapshot data evaluation

The PEG molecule in each simulation snapshot was analyzed for a series of parameters. The following geometric parameters were recorded: dihedral angle between adjacent oxygen atoms (“CC-dihedral”), dihedral angle between adjacent oxygen and carbon atoms (“OC-dihedral”), bond angles (“COC”-, “OCC”-, “CCO”-, and “HCH”-angle), bond length (“OC”-, “CC”-, and “CH”-bond length). Additionally, H-bonds (total number, number donated, number accepted, and total energy), surface characteristics (solvent accessible surface (“SAS”) of CH-groups, O-atoms, and OH-groups), and solvent accessible volume (“SAV”, 1.4Å probe radius) were measured.

### Reference structure generation

Recently published crystallographic data of a PEG molecule [22] was used to build reference structures. The geometric parameters (dihedral angles, bond angles, bond lengths) of the crystallographic record were measured and PEG-molecules with 6 to 81 subunits constructed accordingly. Surface characteristics and solvent accessible volume of these molecules were measured as described for the MD snapshots.

### Data evaluation

Data evaluation was performed using Matlab^®;^ (The Mathworks^™^, Natick, ME, USA), version 2010a. All geometric parameters were first analyzed for a trend over simulation time. If no dependency on simulation time was found, the mean value over all snapshots was used. If no dependency on the position within the molecule was found, the mean value across the molecule was used. Finally, if no dependency on PEG-length was found, the mean value over all PEG-length was calculated. To assess the degree of secondary structure formation of the simulated PEG molecules, regions of four and more consecutive CC-dihedral angles within the “gauche-conformation range” of 50° to 100° or -50° to -100° were considered to form a helical structure. The ratio of CC dihedrals forming a helical structure over the total number of CC dihedrals in the molecule was taken as a measure for the helicality of the molecule. Mean helicality over all snapshots were calculated for each PEG-length simulated. Curve fitting was done using the non-linear-least-square method for fitting and the least-average-residual (“LAR”) method for robustness.

### Binodal data generation

Experimentally determined binodal curves were obtained using the approach previously described [26]. In short, a Tecan (Crailsheim, Germany) Freedom EVO^®;^ 200 robotic platform was used to determine phase transition points using a “cloud point method” modified for liquid handling platforms. PEG used were obtained from Sigma-Adrich (Product numbers were: PEG300: 202371 - PEG600: 202401 - PEG1000: 202428 - PEG1500: 81214 ). 40%[w/w] PO_4_ stock solution at pH 7.0 was composed of 11.9 g sodiumdihydrogenphosphate and 28.1 g di-potassiumhydrogenphosphate per 100 g of solution.

### Polarity measurements

Determination of empirical _*ET*_(30)values were done using the solvatochromic dye nile red (Sigma Aldrich, Steinheim) by measuring the shift of the last absorbance maximum between 500 and 650 nm with a Perkin Elmer lambda 35 spectrophotometer [27]. The following PEGs were investigated: PEG 200, PEG 400, PEG 600, PEG 1000 and PEG 1450 (all purchased from Sigma Aldrich, Steinheim as stated in the previous section). Samples were prepared for different molar fractions of PEG in water with the investigated range depending on the PEG molecular weight (0-44.2% for PEG 200, 0-21% for PEG 400, 0-15.8% for PEG 600, 0-7.8% for PEG 1000 and 0-3.3% for PEG 1450). 2 *μl* of a saturated solution of nile red in 75% v/v acetonitrile were added to 1ml of sample solution. Calibration of the _*ET*_(30) scale was done by plotting _*ET*_(30)values of organic solvents (ethanol and different dilutions of ethanol in water, isopropanol, acetone, toluol and octanol) over the wavelength of the absorbance maximum of nile red. _*ET*_(30) values were taken from literature [28]. Data was fitted with a 2nd degree polynomial function.

## Results

### PEG primary structure in solution

**Figure 1:**
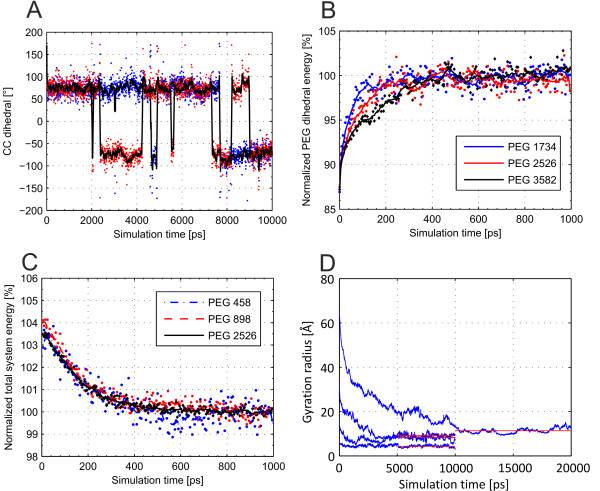
**Structural Dynamics during simulation.** Structural dynamics resulting from the MD simulation. **(a)**: Two dihedral angles of a PEG1162 over simulation time. **(b)**: Dihedral energy of three selected PEGs over simulation time. **(c)**: System total energy over simulation time. Single data points as well as smoothed line calculated over 9 data points are shown. The number of subunits are given in the legends. **(d)**: Radius of gyration over simulation time. Blue lines show raw data of (from top to bottom) PEG2746, PEG1426, PEG766, and PEG326. Red lines show the timespan over which average properties of the polymer molecule were calculated

All simulations were started with a linear PEG molecule, with all geometric parameters set to standard values by the simulation software. Table 2 shows average geometric parameters resulting from the MD simulations in comparison to those obtained from the crystal structure [22]. While tetrahedral angles showed almost no deviation (maximum deviation 0.45%) between the crystal structure and the structure found by our simulations, bond length and dihedral angle differ by 1.4% - 4.1% and 2.0% - 4.1% respectively. CC dihedral and OC distance showed a dependency on the number of PEG subunits. CC dihedral angle increased by 1.02% over the range of PEG subunits investigated herein (see Figure 1-b). The increase in OC bond length was 0.14%.

**Table 2:** **Comparison of geometric parameter between crystal structure of PEG[**[22] and MD simulation results

**Parameter**	**Crystal**	**MDs**
HCH angle [°]	109,01	108,97
CCO angle [°]	109,96	109,87
COC angle [°]	115,64	115,09
OCC angle [°]	109,49	109,87
OC distance [Å]	1,43	1,45-1,452
CC distance [Å]	1,48	1,54
CC dihedral *gauche* [°]	-74,95	-73,43-74,18
OC dihedral *trans* [°]	177,41	170,13

### Secondary structure

**Figure 2:**
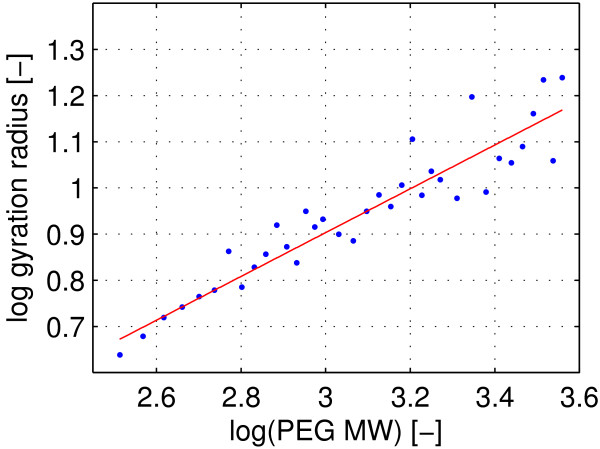
**Gyration radius over PEG molecular weight.** Dots: log _*RG*_plotted over log *PE*_*GMW*_. Line: Linear fit using least average square algorithm. The slope of the linear fit is not significantly different from 0.5

PEG secondary structure was mainly governed by the CC dihedrals settling into gauche conformation. While the average CC dihedral was found in good agreement to the crystal structure (see Table 2), its value increased and the ratio of CC dihedral in gauche conformation decreased with PEG chain length (see Figures 1-b and 1-d). Concurrently, helicality, defined as the ratio of dihedrals partaking in stretches of more than three dihedral of equal conformation, increased with PEG chain length (see Figure 2-c).

**Figure 3:**
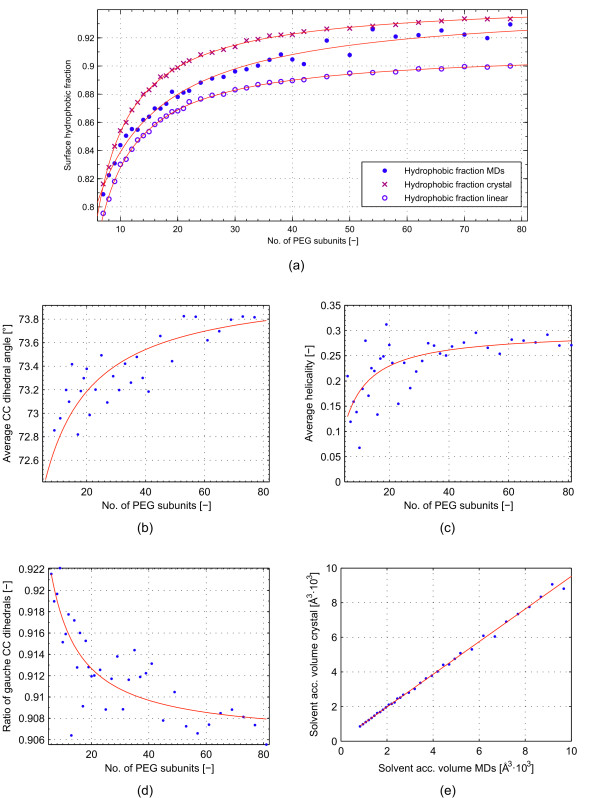
**Surface hydrophobicity and structural attributes.(a)**: Surface hydrophobic fraction of artificially constructed perfectly helical, perfectly linear PEGs and results of the MD simulations over PEG chain length. **(b)-(d)**: factors influencing surface hydrophobicity over PEG chain length with **(b)**: CC dihedral angle, **(c)**: helicality, (d): ratio of CC dihedrals in gauche conformations. (e) solvent accessible volumes of the PEG molecules determined from MD simulations over the corresponding volume of perfectly helical molecules

Figure 3 shows a rendering of a simulation snapshot of a PEG550 (n=11). Helical regions and the surface accessible volume (“SAV”) are noted. In agreement with other publication, the PEG molecule adapts a random-coil conformation with the CC dihedrals preferring a gauche conformation. Stretches of CC dihedral in gauche conformation of equal sign form helical regions within the random coil.

### Tertiary structure

As mentioned in the previous section, the PEG molecules were found to settle into a random-coil conformation in agreement with previously published studies. While the fluctuation of total system energy suggested to have reached an equilibrium after 1 ns of simulation time, values of _*Rg*_reached equilibrium only after 25 ns for the longest PEG molecules. In Figure 2 the log _*RG*_ is plotted over log *PE*_*GMW*_. A linear fit using the least average residual algorithm was calculated and is shown in Figure 2.

The radius of gyration _*Rg*_was found to correlate with the molecular weight with an exponent *v* for $R_{g}\propto M_{w}^{v}$not significantly different from 0.5. This is in agreement with polymer theory for molecules within the tested range of molecular weights [29,30] when ideal chain behavior, or a theta solvent respectively, are assumed. While the exponent *v* was found not to be significantly different from 0.5 the 95% confidence interval accommodates values as high as 0.535 thus also allowing for a divergence towards real-chain behavior. As helical coils were observed as a structural element in the secondary structure, a divergence from the ideal chain model would be reasonable. Longer simulation times might be needed to average out structural fluctuations of _*Rg*_thus narrowing the confidence interval around *v*. However, as mentioned above, as the aim of this study was not to infer from structure to macroscopic solution properties, the fluctuations of _*RG*_ are acceptable within the scope of this work. Solvent accessible volume (“SAV”) of the artificial linear PEG helices and the equivalent average volume of the PEG molecules from MD simulations did not differ significantly. SAVs of simulated PEG molecules were normally distributed around their mean with an average coefficient of variation (“CV”) of 2.4 %. It was concluded, that, while the volumes of PEG molecules in our simulations change dynamically with time, no stable tertiary structures excluding the solvent were formed. While the observed helical regions influenced surface hydrophobicity, they did not significantly change the accessible volume of the molecules.

### Structure dynamics

Figure 1 highlights three aspects of the structural dynamics observed over the course of the simulations. Figure 1-a shows the value of two CC-dihedrals arbitrarily selected from a PEG1162 (n=25) over the entire course of a 5 ns simulation. The two angles are in gauche(-) or gauche(+) conformation in the vast majority of the snapshots. The angles flip irregularly between the (-) and (+) conformation. While the angles are either gauche(-) or gauche(+) most of the time, regions where both angles have the same conformation are more limited. Helical structures form in regions where multiple consecutive CC-dihedrals are of the same conformation. Figure 1-b shows the normalized PEG dihedral energy of three PEGs of different molecular weight over the course of 0.5 ns of simulation. The higher the molecular weight of the PEG, the longer it took to reach equilibrium. Figure 1-c shows the normalized system energy of three PEGs of different molecular weight over the course of 1 ns of simulation. All three simulations show the same trend and reach equilibrium within this time frame. Scattering of the data is lower, the higher the molecular weight of the PEG. Figure 1-d shows the radius of gyration plotted over the simulation time of four exemplary PEG molecules. It can be seen, that the tertiary structure of the PEG reached equilibrium within the simulated timespan. Longer PEG molecules needed longer simulation times. The range over which average properties were calculated is detailed in Figure 2-d. It can be seen, that the tertiary structure is in equilibrium in the time span which was used for the calculation of average PEG properties. Both Figure 1-b and 1-c suggest, that the flexibility of the PEG molecule is dependent on its molecular weight.

### Surface hydrophobicity

**Figure 4:**
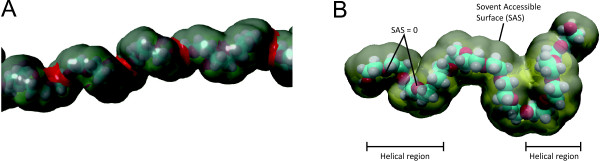
**3D renderings of the resulting structures.(a)**: 3D rendering of a helical region formed during the MD simulation. CH-surfaces are marked gray, O-surfaces are marked red. **(b)**: 3D rendering of a simulation snapshot of a PEG722 including the solvent accessible surface (1.4Å probe radius). Two oxygen atoms having near 0 solvent accessible surface as well as the helical regions are pointed out

As no tertiary structure excluding the solvent was formed during our simulations, it was concluded that the effect of PEG molecules on their surrounding solvent is governed by its solvent accessible surface. Thus, the influence of PEG chain length on the surface characteristics were investigated. Figure 3-a shows the dependency of the surface hydrophobic fraction on the number of PEG subunits. Surface hydrophobic fraction was defined as the solvent accessible surface of CH-groups in relation to the total surface. Three dataset are compared in this figure: first,the results from the MD simulations; second, hypothetical, entirely helical PEG molecules constructed with the geometric parameters obtained from the crystal structure as describe in the section ‘Reference structure generation’, and third, hypothetical, entirely linear PEG molecules, constructed in the same way as the helical structures, but with dihedral angles all set to 180°. Three effects are discernible. First, there is an offset between surface hydrophobicity of the helical and the linear structure. It was concluded, that the degree of surface hydrophobicity is strongly influenced by the secondary structure of the PEG molecule. Within helical regions, oxygen atoms face inwards, away from the solvent, while CH-groups are turned towards the solvent, thus increasing surface hydrophobicity (see Figure 4-a).

It should be noted that due to the compaction of the overall structure, both the hydrophobic and the hydrophilic surface area decrease in the process of helix formation. However, hydrophilic surface area decreases more than the hydrophobic surface area thus making the overall surface more hydrophobic. Second, all three curves show a dependency of the surface hydrophobic fraction on the number of PEG subunits. This effect is mostly independent of the secondary structure. By comparing the hydrophobicity of the end- and mid-groups of the various PEGs, the effect could be ascribed to the “dilution” of the end group with increasing PEG chain length. The end groups are less hydrophobic than the repetitive units in the middle of the molecule. Thus, surface hydrophobicity increases the more of these repetitive units exist. Third, surface hydrophobic fraction of the MD simulations are generally below those of the entirely helical structures. At higher numbers of subunits, the values start to converge. To explain this trend, several underlying effects need to be looked at. As helicality of a PEG molecule has a major effect on its surface hydrophobicity, and helicality increases with number of PEG subunits (see Figure 3-c), it is reasonable that MD simulation results close in on the surface characteristics of a perfectly helical molecule with increasing molecular weight. While helicality increases, helix angle also increases (Figure 3-b) and fraction of CC dihedrals in gauche conformation decreases (Figure 3-d), with both effects decreasing surface hydrophobicity. Additionally, as shown in Figure 4-b, during the MD simulations, temporary structures can be formed that hardly change the solvent accessible volume but strongly reduce the solvent accessible surface of certain parts of the molecules. These regions were found randomly distributed over time and place in the MD simulations and contribute to a decrease in surface hydrophobicity as they only affect mid-groups. The relation of surface hydrophobicity to molecular weight were the result of the combination of all the underlying effects mentioned above.

### PEG-solvent interaction

**Figure 5:**
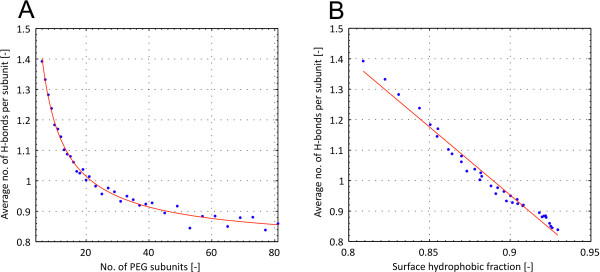
**Relationship between surface hydrophobicity and H-bonding.** Average number of H-bonds per subunits over number of subunits (top) and surface hydrophobic fraction (bottom)

To quantify the influence of the change in surface hydrophobicity on the interaction of the PEG molecule with their surrounding solvent, average number of H-bonds per PEG subunit were plotted over PEG chain length and surface hydrophobicity in Figure 5 Average number of H-bond per subunit decreased with increasing PEG chain length in the form of a power function. A linear correlation between average H-bonds per subunit and surface hydrophobic fraction was found with ^*R*2^=0.963.

**Figure 6:**
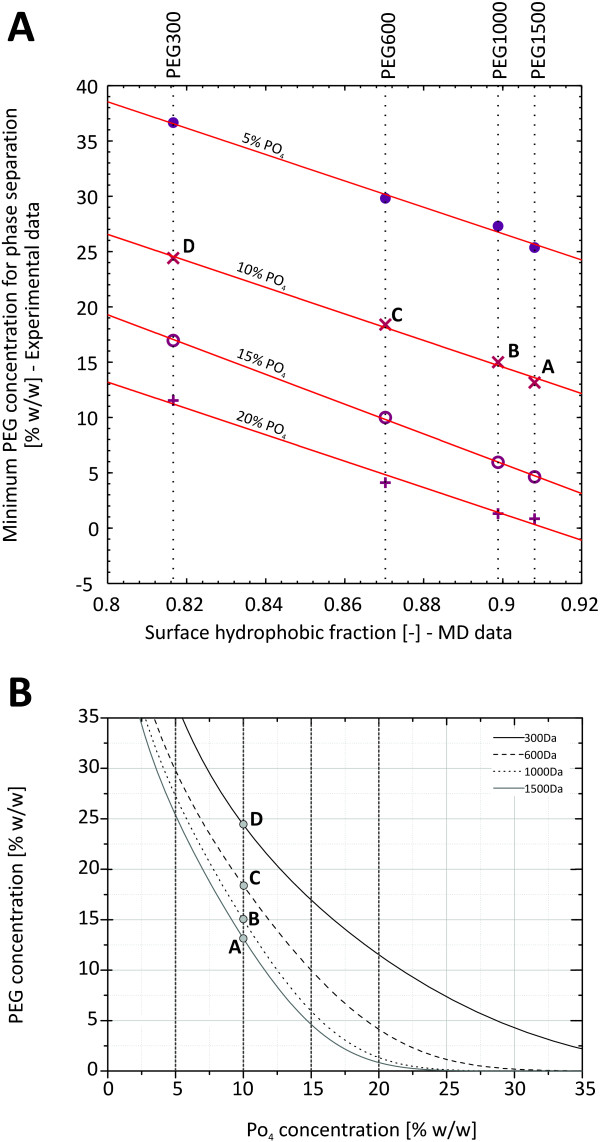
**Relationship between surface hydrophobicity and two phase formation.** Experimentally determined PEG concentration needed for two-phase formation of four different PEG molecular weights at four concentrations of PO_4_ plotted over the surface hydrophobic fraction of the corresponding PEG molecules determined from MD simulations. Phase formation in the presence: ●5% PO_4_, ×10% PO_4_, ∘15% PO_4_, + 20% PO_4_. **B:** Experimentally determined binodals of PEG-PO_4_ ATPSystems. Dashed lines represent the levels of PO_4_ concentration at which the minimum concentration of PEG needed for phase separation was determined. The data point labeled “A”, “B”, “C”, and “D” are the same points on the binodals in both subfigures

Binodal curves of PEG300, PEG600, PEG1000 and PEG1500 in combination with PO_4_ at pH 7.0 were determined experimentally. The least PEG concentration needed for two-phase formation at four different concentrations of PO_4_ were plotted against the surface hydrophobic fraction of the corresponding PEG determined in the MD simulations (Figure 6). Linear correlations with an average ^*R*2^ of 0.994 were found for all four PO_4_ concentrations. The good correlation between experimental and simulation data suggests that the taken simulation approach was successful in generating meaningful polymer surface properties.

### Polarity measurements

**Figure 7:**
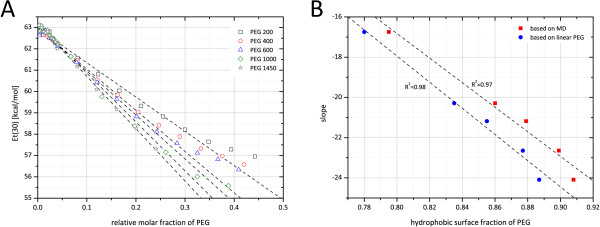
**Relationship between surface hydrophobicity and solvent polarity.A:**_*ET*_(30)values of solutions of PEG of varying molecular weight plotted over the relative mole fraction of PEG calculated as described in the section “Polarity measurements”. The slopes of the linear parts of the plots were used as measure for the polarity of PEG. **B:** The slopes as determined in subfigure **A** plotted over the surface hydrophobicity calculated from MD simulation results of the corresponding PEG molecules

Figure 7-a shows _*ET*_(30)values as an empirical measure for solvent polarity of PEG in an aqueous solution (PEG 200 to 1450) at different molar fractions. Molar fractions of higher PEG molecular weights were corrected relative to PEG 200 to assure comparability of different PEGs (PEG 400 molar fractions were multiplied by 2, molar fractions of PEG 600 by 3 and so on). Polarity decreased with increasing molar fraction of each PEG. PEG with higher molecular weight was more hydrophobic at the same relative molar fraction (meaning that 2 molecules of PEG 200 were less hydrophobic than 1 molecule of PEG 400). With increasing molar fractions the decrease of _*ET*_(30) clearly deviated from linearity. For that reason the slope of the linear region was used as a basis for comparing polarities of different PEGs rather than using the extrapolated intercept with the x-axis. These values showed a linear correlation with hydrophobicity values generated on the basis of PEG structure information as shown in Figure 7-b.

## Discussion

### Validation of rectangular box geometry

**Figure 8:**
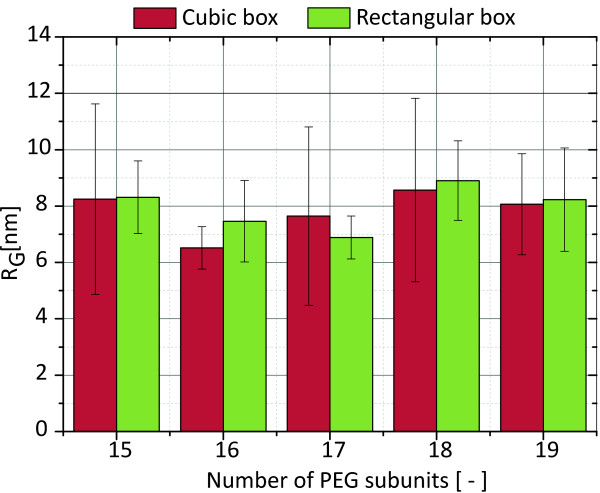
**Comparison of the radius of gyration resulting from a cubic box to the radius of gyration resulting from a rectangular box.** A subset of PEG simulations was run both in a rectangular and in a cubic box. The resulting average radii of gyration fall well within the standard deviations (shown as error bars) of one another

Using a rectangular, non-cubic rather than a cubic box geometry in MD simulation is uncommon and bears certain risks. The reason for choosing such an uncommon box geometry in the simulations presented herein is outlined in the following section. For the PEG molecule to be properly hydrated during the simulation, the simulation has to start with a completely outstretched, linear molecule. Starting with any other structure will result in an improperly hydrated PEG molecule which will, as it sits in a ’local vacuum’, immediately collapse onto itself. This was tested and the resulting radii of gyration were not in agreement with polymer theory. For the longest PEG simulated, approximately 400,000 atoms would be needed to be simulated in a cubic box. With the current computational power available, this approach is unfeasible. The rectangular box geometry used in this study creates the risk of violating the minimum image convention and the risk of creating a systematic error due to interactions of the PEG molecule with itself through crossing a periodic boundary. The simulation software used does not permit to start a simulation that violates the minimum-image convention, or immediately stops the simulation with an error in case cell-rescaling due to pressure coupling leads to a situation where the minimum-image convention is violated (personal communication with the developer of the software). This risk could thus be neglected. The risk of self-interaction was addressed in the following ways. First, videos of the simulations were created and visually checked for signs of significant interaction between parts of the polymer that cross periodic boundaries and parts that do not. We did not find any situation suggesting a systematically occurring interaction. There were instances in which an interaction across a periodic boundary occurred and there were instances in which an interaction not crossing a boundary occurred. There was nothing to suggest that the interactions occurring across a boundary influenced the resulting PEG structure in any way. Second, a subset of simulations were run in a cubic box, for which the simulation box had a ’significantly narrow’ shape and crossing of the periodic boundaries in the initial simulations had been observed. The PEG molecule found in the last snapshot and the surrounding hydration shell were transferred into a cubic box. From this initial structure, 10 ns long simulations were run. Naturally, with a different initial structure, the simulation developed along a slightly different trajectory. However, the new values generated by this simulation (for example the radius of gyration) are well within the standard deviation of the values of the initial simulations. No systematic deviation between the two dataset was observed. This data is plotted in Figure 8.

### Primary & secondary structure

Starting from a completely linear structure with all geometric parameters set to standard values by the importing plug-in, our molecular simulations resulted in average PEG structures with geometric parameters in good agreement with both the crystal structure [22] and previous MD simulations [17].

While Tasaki *et al.*[17] found a ratio of gauche(+) to gauche(-) of 0.33 to 0.67 for the CC dihedral in their 0.5-2ns long simulations, we found equal distribution of the two conformations. As there is no molecular constraint that would lead to an uneven distribution of the conformation, we conclude that the chosen simulation time of 5 ns is sufficient to yield a representative, average structure. System total energy reaching equilibrium within 1 ns supports this conclusion.

A clear dependency of CC dihedral and the OC distance on PEG chain length was found. The increase in CC dihedral (1.02%) was significantly larger than the increase in OC bond length (0.12%). While the dependency was obvious, we consider the degree of change of OC bond length too low to have a significant effect on the resulting surface characteristics. The reason for this dependency remains unclear.

In a recently published paper, Winger *et al.*[18] investigated the influence of force-fields on the resulting structure of a modified PEG molecule. They concluded that the development of a helical structure of the simulated PEG molecule in aqueous solution is dependent on the force-field used. Improper force fields or simulations in vacuo resulted in the PEG molecule collapsing into a random coil. In our study, PEG molecules formed helical structures and stayed elongated. The resulting geometric parameters are in good agreement with the results from previous MD simulations [17] as well as crystallographic data [22] and structural data from other sources (as summarized in [21]). We thus conclude, that the amber03 force-field in concert with the employed parametrization algorithm is well suited to run MD simulations of PEG molecules.

### Structure dynamics

Structure dynamics were looked at in terms of time to reach equilibrium of total system energy, scattering of total system energy and time to reach dihedral energy equilibrium. The general conclusion drawn from these observations was, that longer PEG chains are less flexible. It was further concluded, that the increase in helicality with increasing PEG chain length is a direct consequence of the decreased flexibility. Helical regions are less prone to be broken up if the structure is changing less dynamically. Tasaki *et al.*[17] concluded that their simulation times (0.5-2 ns) were not sufficient to reach an equilibrium. In contrast, system total energy in our simulations reached equilibrium within 1 ns of simulations. We conclude, that simulation times of 1 ns are sufficient when using the simulation protocol employed herein and when the surface of the molecule is the target of the investigation. Reaching an equilibrium tertiary structure, measured as reaching an equilibrium in the radius of gyration, needed significantly longer simulations times as high as 25ns for the largest molecules investigated. Lee *et. al* in 2009 [13] used a different modeling approach to realize simulation times of up to 800 ns in order to investigate PEG tertiary structure and its hydrodynamic properties in water. Such long simulation times are currently outside the reach of all-atom MD simulations. Again, it should be pointed out that the focus of this study was on the secondary structure and PEG solvent interactions.

In 1970 Koenig *et. al* concluded from Raman spectroscopy studies that PEG molecules display a more highly ordered structure in water than in methanol. They hypothesized that the helical nature of the solid state is partly retained in the solubilized state [31]. This was also discussed by Devanand *et. al*[32] and is supported by the results presented herein. While the radius of gyration observed did not support a large deviation from ideal chain behavior, helical regions were found to form within the molecule.

### Surface hydrophobicity

Surface hydrophobicity of the PEG molecules was looked at in terms of surface contribution of CH groups. Two main effects were identified. First, formation of helical regions within the molecule increased the overall hydrophobicity. Second, the effect of the hydrophilic end-group got diluted with increasing PEG chain length. These two effects could explain the overall trend in surface hydrophobicity of the simulated PEG molecules. Additionally, several underlying effects were identified, that influenced surface hydrophobicity and, in part, counteracted one another. For example, both helix angle and overall helicality increased with PEG chain length, with the former decreasing and the latter increasing surface hydrophobicity. While the interplay of the various effects is complex, the overall trend, increasing surface hydrophobicity with increasing PEG chain length showed clearly in our simulations. Zaslavsky *et al.*[33] measured the hydrophobicity of tritium labeled PEG molecules (1.5 to 40 kDa) in terms of distribution in Ficoll-400-dextran-70 ATPS. The distribution was found to be equal within experimental certainty. The authors concluded that the investigated PEG molecules had equal hydrophobicity. While results from the MD simulations described herein suggest an increase in surface hydrophobicity, the smallest PEG molecule investigated by Zaslavsky *et al.* had a MW of 1.5 kDa. At this molecular weight (no. of subunits = 34) we found surface hydrophobicity to become less dependent on PEG chain length. Surface hydrophobicity in our simulations changed most in the range 300Da to 1.1kDa. Thus, it would be most interesting to obtain distribution data for PEG molecules within this range.

### PEG-solvent interaction

In this study, the direct interactions of PEG molecules and the surrounding water molecules via H-bonds were quantified. It was found that the number of H-bonds per PEG subunit is a function of PEG chain length and decreases with increasing number of PEG subunits. This is in agreement with the increased hydrophobic surface fraction and the helical structure formed by the PEG molecule, in which the oxygen atoms face inwards and are thus excluded from interactions with the solvent. Tasaki *et al.*[17] discussed the average number of water molecules associated with the PEG molecule. The association of water molecules with PEG was determined by a water density function around the PEG molecule and found to be 2.9. There are several studies in which water associated with PEG molecules was quantified, the results ranging from 1 to 5 water molecules per PEG subunit. Our results are at the lower end of this range. However, it should be noted, that the interaction via H-bonds quantified here does not necessarily translate directly into the association measured in the before mentioned studies, as the nature of this association remains unclear and is governed by proximity rather than direct interaction.

In general, H-bond formation is an exothermic process. Less formation of H-bond with increasing PEG chain length might create the need for a higher ratio of order water structure around the PEG molecule, which is an entropically unfavorable process. This might have implications on the effect PEG molecules have on other dissolved entities or its behavior on aqueous two-phase systems. A direct correlation of the surface hydrophobic fraction to the experimentally determined PEG concentration needed for two-phase formation in the presence of fixed concentrations of PO_4_ was found. We concluded that the simulation results are a good representation of the actual behavior of PEG molecules in solution and that the hydrophobic character of the molecule might be a driver of phase separation in PEG ATPSs. Further investigations will be based on this approach focusing on phase formation in PEG-PO_4_ ATPSs.

While this article focuses on ATPSs, it should be noted that the results might also be applicable to protein precipitation by PEG. Both its structure and thus the space occupied by the PEG molecule in the solvent [1,34] as well as PEG solvent interactions and thus its influence on solvation energy of the protein might be associated with protein solubility. This will be the scope of following investigations.

### _*ET*_(30) versus relative surface hydrophobicity

To approve the calculation of relative hydrophobicity of PEG based on structural information, we compared _*ET*_(30) as an empirical measure for solvent polarity with calculation results based on PEG structure information. In accordance with theory, PEG solutions became more hydrophobic with increasing molar fraction of PEG, indicating that PEG itself is less polar than water (see Figure 7-a). Over a wide range of PEG concentrations this relationships was found to be linear, at higher PEG concentrations further addition of PEG led to an disproportional decrease of _*ET*_(30). For that reason the slope for the linear part was used as a measure for PEG polarity and correlated with relative PEG hydrophobicity based surface contributions of unpolar −*CH* and polar −*O*− and −*OH* groups of 1) linear PEG molecules and 2) partly helical PEG molecules from MD simulations. In both cases a linear correlation was obtained with similar ^*R*2^values (see Figure 7-b), indicating that the structure-based hydrophobicity measure was suitable to describe changes in PEG hydrophobicity. Nevertheless, the experimental data could not be used to differentiate between linear and partly helical PEG structures.

## Conclusion

In this study, the validity of using the amber03 force field in combination with the AutoSMILES self parameterizing algorithm for MD simulations of PEG was confirmed. MD simulations were run on a series of PEG molecules ranging in molecular weight from 300 Da to 3500 Da. 3D data from these simulations were found in good agreement with recently published crystallographic data and published MD simulation results. It was found that PEG chain length has a major influence on the surface characteristics and solvent interaction of PEG. Surface hydrophobicity values derived from these simulations could be correlated to experimentally determined minimum PEG concentrations needed to establish two-phase systems in the presence of PO_4_. Surface hydrophobicity values from MD simulations correlated linearly with solution polarity experimentally determined via a solvatochromic dye. This work forms the basis for conducting further MD studies on phase behavior of PEG as well as simulations including both PEG and proteins such as proteins in ATPSs or simulations of PEGylated proteins.

## Competing interests

The author(s) declare that they have no competing interests.

## Author’s contributions

SAO conducted the simulations and the experimental work on binodal curve, the experimental evaluations and the preparation of the manuscript. FD supervised the simulations and their evaluation, helped in interpreting the results and in devising the experiments. FD helped in preparing the manuscript and conducted the experiments on solvent polarity. JH provided general guidance on the experiments and their interpretation and helped revising the manuscript. All authors read and approved the final manuscript.

## References

[B1] Atha DH Ingham KC Mechanism of precipitation of proteins by polyethylene glycols. Analysis in terms of excluded volume J biol Chem 1981 256 2323 12108 17 7298647

[B2] Shulgin IL Ruckenstein E Preferential hydration and solubility of proteins in aqueous solutions of polyethylene glycol Biophys chem 2006 120 3 188 98 10.1016/j.bpc.2005.11.010 16377069

[B3] Albertsson PÅ Particle fractionation in liquid two-phase systems The composition of some phase systems and the behavior of some model particles in them application to the isolation of cell walls from microorganisms Biochim Biophys Acta 1958 27 378 395 13522737 10.1016/0006-3002(58)90345-7

[B4] Hatti-Kaul R Aqueous two-phase systems - A general overview Mol Biotechnol 2001 19 3 269 277 10.1385/MB:19:3:269 11721623

[B5] Hansson UB Wingren C Separation of antibodies by liquid-liquid aqueous partition and by liquid-liquid partition chromatography Sep Purif Methods 1998 27 2 169 211 10.1080/03602549809351640

[B6] Huddleston JG Lyddiatt A Aqueous 2-Phase Systems in Biochemical Recovery - Systematic Analysis Design, and Implementation of Practical Processes for the Recovery of Proteins Appl Biochem Biotechnol 1990 26 3 249 279 10.1007/BF02921506

[B7] Sunasara KM Xia F Gronke RS Cramer SM Application of hydrophobic interaction displacement chromatography for an industrial protein purification Biotechnol and Bioeng 2003 82 3 330 9 10.1002/bit.10582 12599260

[B8] Gabizon A Catane R Uziely B Kaufman B Safra T Cohen R Martin F Huang A Barenholz Y Prolonged circulation time and enhanced accumulation in malignant exudates of doxorubicin encapsulated in polyethylene-glycol coated liposomes Cancer res 1994 54 4 987 92 8313389

[B9] Gregoriadis G Engineering liposomes for drug delivery: progress and problems Trends in Biotechnol 1995 13 1212 527 537 10.1016/S0167-7799(00)89017-4 8595139

[B10] Greenwald RB Choe YH McGuire J Conover CD Effective drug delivery by PEGylated drug conjugates Adv drug delivery rev 2003 55 2 217 50 10.1016/S0169-409X(02)00180-1 12564978

[B11] Harris JM Chess RB Effect of pegylation on pharmaceuticals Nat rev Drug discovery 2003 2 3 214 21 10.1038/nrd1033 12612647

[B12] Dismer F Hubbuch J 3D structure-based protein retention prediction for ion-exchange chromatography J of Chromatogr A 2010 1217 8 1343 53 10.1016/j.chroma.2009.12.061 20089254

[B13] Lee H Venable RM Mackerell AD Pastor RW Molecular dynamics studies of polyethylene oxide and polyethylene glycol: hydrodynamic radius and shape anisotropy Biophys j 2008 95 4 1590 9 10.1529/biophysj.108.133025 18456821 PMC2483782

[B14] Vorobyov I Anisimov VM Greene S Venable RM Moser A Pastor RW MacKerell AD Additive and Classical Drude Polarizable Force Fields for Linear and Cyclic Ethers J Chem Theory Comput 2007 3 3 1120 1133 10.1021/ct600350s 26627431

[B15] Lee H AHd Vries Marrink SJ Pastor A coarse-grained model for polyethylene oxide and polyethylene glycol: conformation and hydrodynamicsj of phys Chem B 2009 113 40 13186 94 10.1021/jp9058966 19754083 PMC2937831

[B16] Borodin O Bedrov D Smith GD A Molecular Dynamics Simulation Study of Polymer Dynamics in Aqueous Poly(ethylene oxide) Solutions Macromolecules 2001 34 16 5687 5693 10.1021/ma010038b

[B17] Tasaki K Poly(oxyethylene)-Water Interactions : A Molecular Dynamics Study J Am Chem Soc 1996 118 8459 8469 10.1021/ja951005c

[B18] Winger M de Vries AH van Gunsteren WF Force-field dependence of the conformational properties of α,ω-dimethoxypolyethylene glycol Mol Physics 2009 107 13 1313 1321 10.1080/00268970902794826

[B19] Wang J Wolf RM Caldwell JW Kollman Pa Case Da Development and testing of a general amber force field J comput chem 2004 25 9 1157 74 10.1002/jcc.20035 15116359

[B20] Jakalian A Jack DB Bayly CI Fast, efficient generation of high-quality atomic charges. AM1-BCC model: II. Parameterization and validation J comput chem 2002 23 1623 41 10.1002/jcc.10128 12395429

[B21] Seebach D Zass E Schweizer WB Thompson AJ French A Davis BG Kyd G Bruno IJ Polymer Backbone Conformation-A Challenging Task for Database Information Retrieval Angewandte Chemie-Int Ed 2009 48 51 9596 9598 10.1002/anie.200904422 19890923

[B22] French AC Thompson AL Davis BG High-Purity Discrete PEG-Oligomer Crystals Allow Structural Insight Angewandte Chemie-Int ed 2009 48 7 1248 1252 10.1002/anie.200804623 19142918

[B23] Krieger E Koraimann G Vriend G Increasing the precision of comparative models with YASARA NOVA–a self-parameterizing force field Proteins 2002 47 3 393 402 10.1002/prot.10104 11948792

[B24] Essmann U Perera L Berkowitz ML Darden T Lee H Pedersen LG A smooth particle mesh Ewald method J Chem Phys 1995 103 19 8577 10.1063/1.470117

[B25] Cornell WD Cieplak P Bayly CI Gould IR Merz KM Ferguson DM Spellmeyer DC Fox T Caldwell JW Kollman PA A Second Generation Force Field for the Simulation of Proteins, Nucleic Acids, and Organic Molecules J Am Chem Soc 1995 117 1919 5179 5197

[B26] Oelmeier SA Dismer F Hubbuch J Application of an aqueous two-phase systems high-throughput screening method to evaluate mAb HCP separation Biotechnol and Bioeng 2010 108 69 81 10.1002/bit.22900 20717969

[B27] Sackett DL Wolff J Nile Red as a Polarity-Sensitive Fluorescent-Probe of Hydrophobic Protein Surfaces Anal Biochem 1987 167 2 228 234 10.1016/0003-2697(87)90157-6 3442318

[B28] Dimroth K Bohlmann F Reichard C Siepmann T Über Pyridinium-N-Phenol-Betaine Und Ihre Verwendung Zur Charakterisierung Der Polarität Von Lösungsmitteln Annalen Der Chemie-Justus Liebig 1963 661 1 37 10.1002/jlac.19636610102

[B29] Tanford C Physical Chemistry of Macromolecules 1961 John Wiley & Sons, Hoboken, New Jersey, USA

[B30] Doi M Edwards SF The Theory of Polymer Dynamics 1986 Claredon Press: Clarendon Press, Oxford University Press, New York,USA

[B31] Koenig JL Angood AC Raman spectra of poly(ethylene glycols) in solution Journal of Polymer Science Part A-2: Polymer Physics 1970 8 10 1787 1796 10.1002/pol.1970.160081013

[B32] Devanand K Selser J Asymptotic behavior and long-range interactions in aqueous solutions of poly (ethylene oxide) Macromolecules 1991 24 22 5943 5947 10.1021/ma00022a008

[B33] Zaslavsky B Baevskii A Rogozhin S Gedrovich A Shishkov A Gasanov A Masimov A Relative hydrophobicity of synthetic macromolecules I. Polyethylene glycol, polyacrylamide and polyvinylpyrrolidone J Chromatogr A 1984 285 63 68

[B34] Arakawa T Timasheff SN Mechanism of poly(ethylene glycol) interaction with proteins Biochemistry 1985 24 2424 6756 62 4074726 10.1021/bi00345a005

[B35] Sorin EJ Pande VS Exploring the Helix-Coil Transition via All-Atom Equilibrium Ensemble Simulations Bioph J 2005 88 4 2472 2493 10.1529/biophysj.104.051938 PMC1305346 15665128

[B36] Duan Y Wu C Chowdhury S Lee MC Xiong G Zhang W Yang R Cieplak P Luo R Lee T A point-charge force field for molecular mechanics simulations of proteins based on condensed-phase quantum mechanical calculations J comput chem 2003 24 1616 1999 2012 14531054 10.1002/jcc.10349

[B37] Krieger E Darden T Nabuurs SB Finkelstein A Vriend G Making optimal use of empirical energy functions: force-field parameterization in crystal space Proteins 2004 57 4 678 83 10.1002/prot.20251 15390263

[B38] Berendsen HJC Postma JPM Gunsteren WFv DiNola A Haak JR Molecular dynamics with coupling to an external bath Journal Chem Phys 1984 81 8 3684 10.1063/1.448118

